# Impacts of Outdoor Air Pollution on Human Semen Quality: A Meta-Analysis and Systematic Review

**DOI:** 10.1155/2020/7528901

**Published:** 2020-04-28

**Authors:** Jianzhong Zhang, Zhonglin Cai, Chengquan Ma, Jian Xiong, Hongjun Li

**Affiliations:** Department of Urology, Peking Union Medical College Hospital, Peking Union Medical College, Chinese Academy of Medical Sciences, Beijing 100730, China

## Abstract

**Introduction:**

Several studies have explored the association between outdoor air pollution and semen quality. However, the results were inconsistent. We performed the current meta-analysis to evaluate the role of outdoor air pollution in semen quality. *Material and Methods*. Databases including PubMed, Web of Science, and Embase were searched to identify relevant studies. Relative data in participants under higher exposure and lower exposure to air pollution were extracted. Pooled weighted mean differences (WMDs) with corresponding 95% confidence intervals (CIs) were utilized to assess the effects of outdoor air pollution on semen quality. In addition, trial sequential analyses (TSAs) were performed to obtain a more comprehensive assessment of analyses.

**Results:**

A total of 11 studies with 4562 males were enrolled in the current meta-analysis. Higher air pollution levels were associated with significant decreases in semen volume (WMD: -0.16, 95% CI: -0.27 to -0.05), sperm concentration (WMD: -5.52, 95% CI: -9.88 to -1.16), progressive motility (WMD: -6.23, 95% CI: -11.64 to -0.81), total motility (WMD: -7.65, 95% CI: -14.09 to -1.20), and normal sperm morphology rate (WMD: -3.71, 95% CI: -5.59 to -1.82). In addition, the DNA fragmentation index significantly increased (WMD: 4.11, 95% CI: 1.94 to 6.29).

**Conclusions:**

Air pollution is associated with decreased semen volume, sperm concentration, motility, and normal morphology rate.

## 1. Introduction

According to the definition of the International Organization for Standardization (ISO), air pollution usually refers to the phenomenon that harmful or excessive quantities of substances enter the atmosphere due to human activities or natural processes. When the pollutants accumulate to enough concentration and sustained for enough time, air pollution will significantly impair the health of human beings. It can result in various diseases including cardiovascular and lung diseases, neurologic disorders, and infertility [1–5]. Recently, various studies have explored the effects of air pollution on male fertility [6, 7].

Human semen quality has been degraded for decades. Several studies have demonstrated that exposure to toxicants or air pollutants, electromagnetic waves from cell phones, obesity, drinking, smoking, psychological stress hypertension, and diabetes can be potential causes of this degradation [6, 8–14]. Considering the large number of affected populations, outdoor air pollution has become the hotspot recently. However, the specific role of air pollution in semen quality remains unclear. Epidemiologic studies have demonstrated nonsignificant or contrary results. Several studies demonstrated that air pollution can significantly reduce the sperm concentration [15–18] and total sperm count [16–18], but several studies did not show significant results. Concerning the sperm motility, air pollution was reported associated with decreased progressive sperm motility [15, 16, 19–21] and total sperm motility [16, 19–22]. However, some other studies did not demonstrate significant results.

Based on the data in the previous published studies, the current meta-analysis was performed to explore the overall impacts of air pollution on semen quality.

## 2. Materials and Methods

This study was strictly reported based on the PRISMA (Preferred Reporting Items for Systematic Review and Meta-analyses) statement [23]. The protocol of the present study was described previously [24] and registered in the international prospective register of systematic reviews (registration number CRD42019126060). We used the same research methods in the current study.

The quality of the enrolled studies was evaluated by Newcastle-Ottawa Scale (NOS) star system (range, 0 to 9 stars), which focuses on three broad perspectives: the selection of the study groups, the comparability of the groups, and the ascertainment of either the exposure or outcome of interest. The number of stars is positively associated with the quality of the study. Overall, the enrolled studies rated from 6 to 9 stars (Table 1).

## 3. Results

### 3.1. Basic Characteristics of the Enrolled Studies

**Figure 1:**
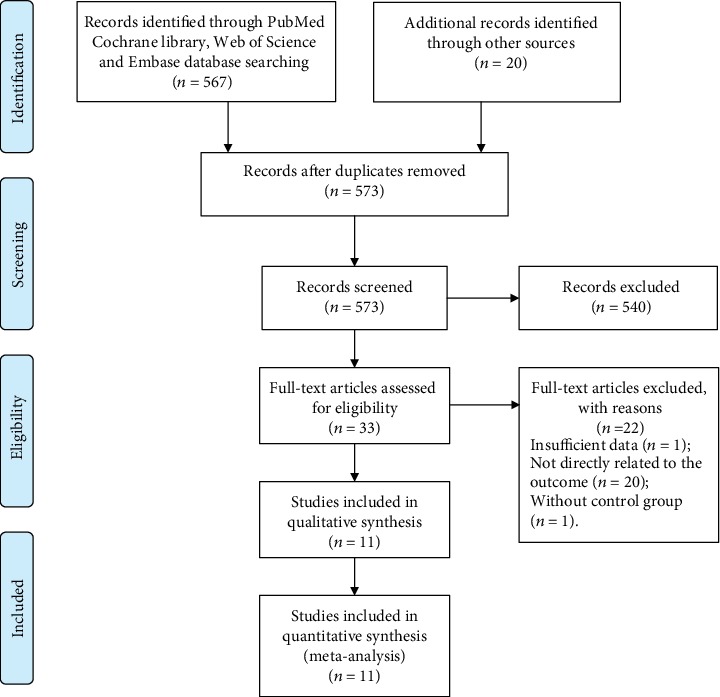
Flow diagram of the study selection process.

The study selection process was shown in Figure 1. In total, eleven studies with 4652 males met the inclusion criteria and were enrolled in the current meta-analysis [15–22, 25–27]. Notably, the outdoor air pollutants varied between the included studies. Four studies explored the role of traffic pollutants in male fertility and did not analyze the composition of the air pollutants. Among the 11 enrolled studies, nine were cross-sectional studies while the other two were longitudinal studies. Seven articles mainly focused on Caucasians and four focused on Asians. Participants were divided into different groups based on the extent of exposure to air pollution. Five, two, and four studies were grouped together according to the location, climate, and working conditions of the participants, respectively. Details of the aforementioned data are listed in Table 1.

### 3.2. The Effects of Outdoor Air Pollution on Sperm Parameters

**Figure 2:**
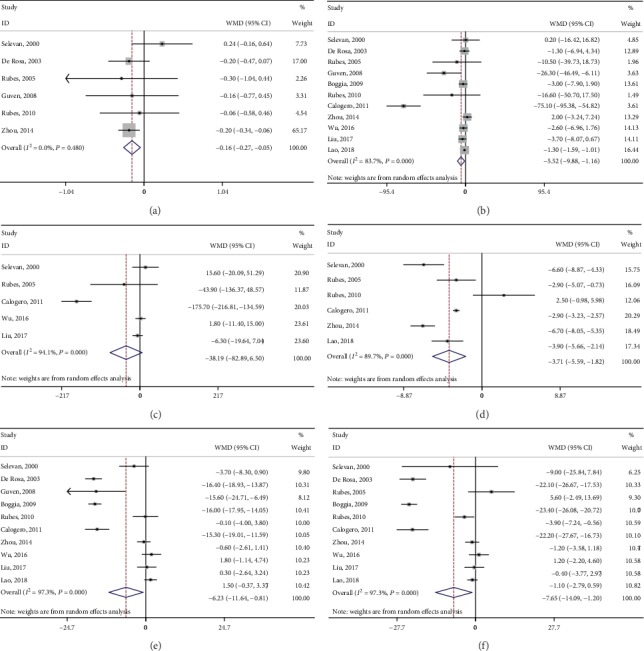
Forest plots of merged analyses of effects on sperm parameters by outdoor air pollution. (a–e) Forests plots of merged analyses of semen volume, sperm concentration, total sperm count, normal morphology rate, progressive motility, and total sperm motility, respectively.

All eleven studies reported the role of outdoor air pollution in sperm concentration. Among them, six [15, 19, 20, 22, 25, 26] and five [16–19, 25] studies further explored the alterations in semen volume and total sperm count, respectively. The results indicated that higher air pollution levels were associated with significant decreases in semen volume (WMD: -0.16, 95% CI: -0.27 to -0.05) (Figure 2(a)) and sperm concentration (WMD: -5.52, 95% CI: -9.88 to -1.16) (Figure 2(b)). Notably, the decrease in total sperm count, which was obtained by multiplying semen volume by sperm concentration, was not significant (WMD: -38.19, 95% CI: -82.89 to 6.50) (Figure 2(c)). This may have partly resulting from the limited sample size.

**Figure 3:**
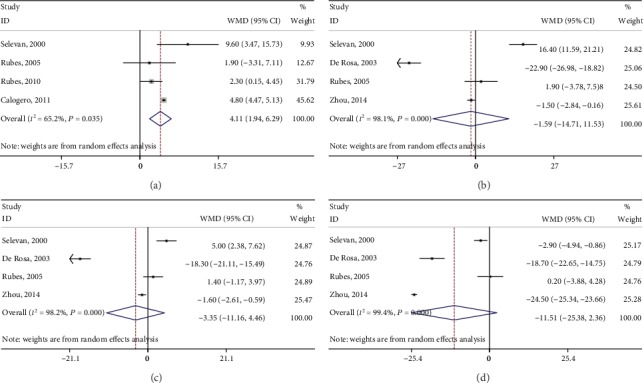
Forest plots of merged analyses of effects on DFI and CASA measures by outdoor air pollution. (a–d) Forests plots of merged analyses of DFI, VCL, VSL, and LIN, respectively.

Six studies explored the association between air pollution and normal sperm morphology rate [16, 19, 22, 25–27]. The pooled results demonstrated a significant decrease in normal morphology (WMD: -3.71, 95% CI: -5.59 to -1.82) (Figure 2(d)). Ten studies explored the association between outdoor air pollution and sperm motility [15–22, 26, 27]. The results indicated that air pollution was associated with significant decreases in progressive motility (WMD: -6.23, 95% CI: -11.64 to -0.81) (Figure 2(e)) and total motility (WMD: -7.65, 95% CI: -14.09 to -1.20) (Figure 2(f)). In addition, the DNA fragmentation index significantly increased based on the pooled result from four studies [16, 19, 22, 25] (WMD: 4.11, 95% CI: 1.94 to 6.29) (Figure 3(a)). Details of the aforementioned information of each enrolled study are listed in Table 2.

CASA measures were provided in four studies [19, 20, 25, 26], and our meta-analysis demonstrated nonsignificant decreases in VCL (WMD: -1.59, 95% CI: -14.71 to 11.53) (Figure 3(b)), VSL (WMD: -3.35, 95% CI: -11.16 to 4.46) (Figure 3(c)), and LIN (WMD: -11.51, 95% CI: -25.38 to 2.36) (Figure 3(d)). Detailed information concerning CASA measures of each enrolled study is listed in Table 3.

### 3.3. Trial Sequential Analysis Results

**Figure 4:**
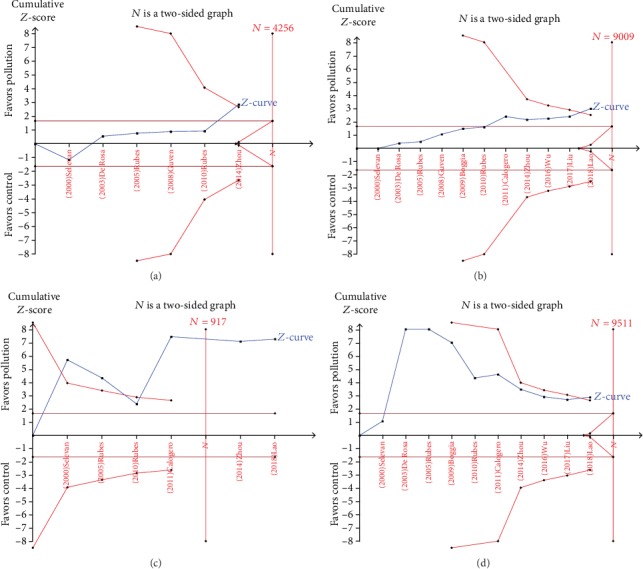
Trial sequential analysis of the effects of TST. (a–d) TSA of semen volume, sperm concentration, normal morphology rate, and total sperm motility.

The TSA results indicated sufficient evidence that outdoor air pollution reduced semen volume (Figure 4(a)), sperm concentration (Figure 4(b)), normal morphology rate (Figure 4(c)), and total motility (Figure 4(d)). However, analysis of progressive motility showed a negative result, indicating that inaccuracy might exist (data not shown). Further studies are required to explore the role of outdoor air pollution in sperm progressive motility.

### 3.4. Sensitivity Analysis

The influence of individual studies on the pooled WMDs was evaluated by sensitivity analyses (Figure S1). No significant alterations in pooled WMDs were observed after any single study was omitted, demonstrating that the results were robust.

### 3.5. Publication Bias

The results of Egger's linear regression tests demonstrated no potential publication bias of the enrolled studies (Semen volume: *P* = 0.433; sperm concentration: *P* = 0.124; total sperm count: *P* = 0.372; progressive motility: *P* = 0.854; total motility: *P* = 0.495; normal morphology rate: *P* = 0.528; DFI: *P* = 0.689; VCL: *P* = 0.984; VSL: *P* = 0.795; and LIN: *P* = 0.260). In addition, evidence of obvious asymmetry was not found in the funnel plots (Figure S2).

## 4. Discussion

Testicular function and sperm development can be affected by exposure to various environmental pollutants, including isoflavones, heavy metals, chlorination disinfection by-products in water, organic solvents, and particulate air pollution [14]. Recently, various studies focused on other harmful environment urban factors, especially the electromagnetic waves from cell phones and stations, can also decrease semen quality and their negative influence cannot be objectively separated from the other environmental pollutants [12, 13].

The effect of pollutants on sperm quality could be evaluated in humans or in laboratory animals. Several animal studies have been performed to investigate the negative effects of air pollution on semen parameters. Prenatal exposure to diesel exhaust has been associated with a significant reduction in daily sperm production, multinucleated giant cells in the seminiferous tubules, partial vacuolation of the seminiferous tubules, and elevated follicle-stimulating hormone receptor (FSHR) mRNA expression in mice [28]. The biological mechanisms of the effects of air pollution on semen quality remain uncertain, and relevant research is limited. One possible mechanism is disorder in the hypothalamic pituitary axis. Particulate matter, i.e., microscopic solid or liquid matter suspended in the atmosphere of Earth, can carry multiple trace elements and polycyclic aromatic hydrocarbons (PAHs). PAHs are a group of compounds that include several endocrine disruptors and can influence sexual hormones by interfering with the hypothalamic pituitary axis [29]. In addition, PAHs can directly impair spermatogenesis [30]. Several studies have demonstrated that the reactive metabolites of PM_10_ and PM_2.5_ can reach the testes and cause increased mitochondrial dysfunction, DNA fragmentation, and cell apoptosis [30, 31]. O_3_-induced oxidative stress is another possible mechanism. Sperm exist in a balanced physiological environment of reactive oxygen species (ROS) and antioxidants. O_3_ may result in inflammation in the male genital tract and formation of circulating toxic species and ROS. Excessive amounts of ROS can subsequently impair the integrity of the DNA in the sperm nucleus and accelerate the process of sperm apoptosis [32, 33]. Luo et al. demonstrated that gasoline exhaust can cause significant reduction in *α*6-integrin and *β*1-integrin in the rat testes, which may be a cause of decreased semen quality [34].

A large number of epidemiologic studies have explored the associations between outdoor air pollution and semen quality. However, the results were inconsistent. The current meta-analysis was performed to obtain conclusive results by pooling all qualified data. The results indicated that outdoor air pollution can significantly impair semen quality by increasing sperm DFI and decreasing semen volume, sperm concentration, motility, and normal morphology rate.

Notably, although semen volume and sperm concentration significantly decreased in participants with higher exposure to air pollution was revealed, the decrease in total sperm count, which is obtained by multiplying semen volume by sperm concentration, was not significant. There were several causes for the nonsignificant result. First, although most of the enrolled studies (11 studies) focused on the semen concentration, only 5 of them provided total sperm count data. Limited sample size can be one cause for the nonsignificant decrease in total sperm count. Second, the standard deviations of the total sperm count were larger than those of the semen volume and sperm concentration. Based on this, a larger sample size is required to reach statistical significance.

It should be noted that sperm development consists of three different key periods: spermatogenesis, development of sperm motility, and epididymal storage, which correspond to 70-90, 10-14, and 0-9 days before ejaculation, respectively. Several studies have reported relatively short-term effects (10-14 or 0-9 days before ejaculation) of air pollution on semen parameters but the results were inconclusive. Notably, the exposure assessment of most included studies in the current meta-analysis was based on the information from monitoring stations for at least 90 days before semen sampling, which provided information about relatively long-term effects of air pollution. Further animal researches and epidemiologic studies are required to explore the effects of air pollution on different periods of sperm development.

The current study has several strengths: (1) the sample size was relatively larger, which made our results more reliable; (2) sensitivity analyses, Egger's linear regression tests, and funnel plots indicated that there were no low-quality studies or publication bias; and (3) TSAs were first performed in the current meta-analysis and indicated sufficient evidence that outdoor air pollution can reduce semen volume, sperm concentration, normal morphology rate, and total motility. Notably, compared with those in a previous meta-analysis that included 6 studies [35], the cumulative *Z*-curves in the current meta-analysis crossed the trial sequential monitoring boundaries, meaning the total sample size was more than the estimated information size after adding another 5 studies.

Though this study had a relatively large sample size, several limitations should be stressed: (1) the sources and concentration of the air pollutants varied among the enrolled studies, which may increase the heterogeneity between studies and result in potential bias. One reason for this is that pollution levels were different in different regions or seasons, making it difficult to set the same standard. In this meta-analysis, all relevant researches were strictly scanned and most of the studies only provided information about the overall impacts of air pollution. Based on the existing data, the current meta-analysis is aimed at exploring the overall impacts of air pollution on semen quality. (2) The impacts of the single components of the air pollutants were not analyzed because studies provided information concerning single components were limited. Further studies are required to explore the impacts of each component such as SO_2_ and CO. (3) Other harmful environment urban factors, such as water pollution or electromagnetic waves from cell phones and stations, can decrease semen quality, and their negative influence cannot be objectively separated from the negative influence of toxic air components. (4) Most participants enrolled in this study were Caucasians, and relevant data in Africans and Asians were limited and required further study. (5) Only four studies focused on CASA measures with inconsistent conclusions, and more studies are needed to investigate the effects of air pollution on these indicators. (6) Though the results of TSA indicated a firm association between air pollution and decreased semen volume, concentration, progressive motility, and total motility, more high-quality studies are required to offer more individual data.

## 5. Conclusion

Air pollution is associated with decreased semen volume, sperm concentration, motility, and normal morphology rate.

## Figures and Tables

**Table 1:** Basic characteristics of the enrolled studies.

Study	Exposure	Period	Study design	Location	Major ethnicity	Age	Groups	Positive findings	NOS
Selevan, 2000	PM10, TSPs, SO2, CO, Nox	1993-1994	Cross-sectional	Czech	Caucasian	18	Residents in urban or rural areas	Lower progressive motility, total motility, normal morphology rate, and chromatin structure	8
De Rosa, 2003	Traffic pollutants	2000-2002	Cross-sectional	Italy	Caucasian	23-62	Workers at motorways or offices	Lower total motility, progressive motility, concentration, VSL, VCL, LIN, and ALH	6
Rubes, 2005	PM10, PAH, SO2, NOx	1995-1997	Longitudinal	Czech	Caucasian	19-25	Residents in winter and summer	None	9
Guven, 2008	Traffic pollutants	NM	Cross-sectional	Turkey	Caucasian	35.2 ± 6.4; 33.7 ± 6.7	Workers at motorways or offices	Lower normal morphology rate, concentration, progressive motility	6
Boggia, 2009	Traffic pollutants	2000-2004	Cross-sectional	Italy	Caucasian	23-57	Workers at motorways or offices	Lower total motility and progressive motility	6
Rubes, 2010	PM2.5, SO2, NO, CO, O3, PAH, Benzo	2007	Longitudinal	Czech	Caucasian	33.6 ± 5.3	Residents in winter and spring	Lower total motility and DFI	9
Calogero, 2011	Traffic pollutants	NM	Cross-sectional	Italy	Caucasian	20-47	Workers at motorways or offices	Lower normal morphology rate, concentration, total sperm count, progressive motility, and DFI	6
Zhou, 2014	PM10, SO2, NO2	2007	Cross-sectional	China	Asian	20-40	Residents in urban or rural areas	Lower normal morphology rate, VSL, VCL, and VAP	8
Wu, 2016	PM10	2013-2015	Cross-sectional	China	Asian	34.4 ± 5.4	Residents with different PM exposure	Lower concentration and total count	9
Liu, 2017	SO2	2013-2015	Cross-sectional	China	Asian	34.4 ± 5.4	Residents with different PM exposure	Lower total count, concentration, progressive motility, and total motility	9
Lao, 2018	PM2.5	2001, 2004	Cross-sectional	China	Asian	31.9 ± 4.3	Residents with different PM exposure	Lower normal morphology and higher sperm concentration	8

ALH: amplitude of lateral movement of sperm head; DFI: DNA fragmentation index; LIN: linearity of sperm motion; NM: not mentioned; NOS: Newcastle-Ottawa Scale; TSPs: PM-total suspended particulates; VCL: sperm curvilinear velocity; VSL: sperm linear velocity.

**Table 2:** Primary outcomes of the enrolled studies.

Study	Sample size		Semen volume (mL)		Sperm concentration (10^6^/mL)		Total count (10^6^)	
	High	Low	High	Low	High	Low	High	Low
Selevan, 2000	47	162	2.2 ± 1.3	2.0 ± 1.1	60.1 ± 46.7	59.9 ± 64.3	129.1 ± 103.1	113.5 ± 130.7
De Rosa, 2003	85	85	2.5 ± 0.9	2.7 ± 0.9	32.4 ± 22.1	33.7 ± 14.7	NM	NM
Rubes, 2005	36	36	3.0 ± 1.7	3.3 ± 1.5	81.6 ± 42.09	92.1 ± 79.0	234.2 ± 141.1	278.1 ± 245.4
Guven, 2008	38	35	3.2 ± 1.3	3.4 ± 1.4	44.6 ± 36.3	70.9 ± 50.0	NM	NM
Boggia, 2009	100	64	NM	NM	34.3 ± 20.3	37.3 ± 11.7	NM	NM
Rubes, 2010	47	47	3.2 ± 1.3	3.2 ± 1.3	134.2 ± 84.1	150.8 ± 84.6	NM	NM
Calogero, 2011	36	32	NM	NM	24.1 ± 15.4	99.2 ± 56.7	64.9 ± 43.3	240.6 ± 111.4
Zhou, 2014	429	917	2.3 ± 1.1	2.5 ± 1.4	79.4 ± 46.2	77.4 ± 44.6	NM	NM
Wu, 2016	367	349	NM	NM	39.4 ± 29.1	42.0 ± 30.3	104.4 ± 82.9	102.6 ± 96.4
Liu, 2017	370	327	NM	NM	39.4 ± 27.3	43.1 ± 31.1	108.4 ± 82.0	114.7 ± 96.0
Lao, 2018	535	501	NM	NM	40.6 ± 2.5	41.9 ± 2.3	NM	NM

Study	Progressive motility (PR, %)		Total motility (PR + NP, %)		Normal morphology (%)		SCSA-DFI (%)	
	High	Low	High	Low	High	Low	High	Low
Selevan, 2000	32.5 ± 13.2	36.2 ± 17.1	41.6 ± 40.4	50.6 ± 79.6	13.2 ± 6.5	19.8 ± 8.5	28.8 ± 20.4	19.2 ± 12.2
De Rosa, 2003	12.3 ± 11.0	28.7 ± 4.6	34.7 ± 20.2	56.8 ± 7.4	NM	NM	NM	NM
Rubes, 2005	NM	NM	68.3 ± 12.1	62.7 ± 21.6	8.4 ± 2.6	11.3 ± 6.1	15.4 ± 12.6	13.5 ± 9.8
Guven, 2008	54.7 ± 23.6	70.3 ± 15.6	NM	NM	NM	NM	NM	NM
Boggia, 2009	15.0 ± 7.4	31.0 ± 5.3	37.0 ± 11.2	60.4 ± 6.3	NM	NM	NM	NM
Rubes, 2010	58.0 ± 9.9	58.1 ± 9.4	70.5 ± 8.2	74.4 ± 8.3	21.3 ± 9.8	18.8 ± 7.2	12.4 ± 5.8	10.1 ± 4.8
Calogero, 2011	12.4 ± 8.7	27.7 ± 6.9	29.6 ± 12.8	51.8 ± 10.2	17.2 ± 0.8	20.1 ± 0.6	9.3 ± 0.9	4.5 ± 0.4
Zhou, 2014	51.7 ± 17.5	52.3 ± 17.5	69.8 ± 20.9	71.0 ± 20.4	23.5 ± 11.5	30.2 ± 12.3	NM	NM
Wu, 2016	38.8 ± 17.7	37.0 ± 22.1	45.8 ± 20.6	44.6 ± 25.4	NM	NM	NM	NM
Liu, 2017	38.9 ± 19.6	38.6 ± 19.9	45.9 ± 22.5	46.3 ± 22.8	NM	NM	NM	NM
Lao, 2018	48.4 ± 15.4	46.9 ± 15.3	65.3 ± 14.0	66.4 ± 13.7	67.9 ± 15.2	71.8 ± 13.7	NM	NM

NM: not mentioned.

**Table 3:** CASA measures of the enrolled studies.

Study	VCL (*μ*m/s)		VSL (*μ*m/s)		LIN (%)	
	High	Low	High	Low	High	Low
Selevan, 2000	107.8 ± 12.1	91.4 ± 21.7	48.3 ± 7.4	43.3 ± 10.0	44.7 ± 5.6	47.6 ± 8.2
De Rosa, 2003	29.7 ± 18.4	52.6 ± 5.5	16.1 ± 12.0	34.4 ± 5.5	47.1 ± 15.6	65.8 ± 10.1
Rubes, 2005	72.8 ± 11.3	70.9 ± 13.2	36.4 ± 4.7	35.0 ± 6.3	52.4 ± 8.1	52.2 ± 9.5
Zhou, 2014	51.9 ± 12.1	53.4 ± 10.8	32.1 ± 9.0	33.7 ± 8.5	60.4 ± 8.4	84.9 ± 4.2

LIN: linearity of sperm motion; VCL: sperm curvilinear velocity; VSL: sperm linear velocity.

## Data Availability

This is a systematic review and all the data were extracted from the enrolled studies. The original data can be accessed in these studies.

## Supplementary Materials

Supplementary Materials Supplement Materials Figure S1: sensitivity of each included study in this meta-analysis. (A–K) Sensitivity analyses of semen volume, sperm concentration, total sperm count, normal morphology rate, progressive motility, total sperm motility, DFI, VCL, VSL, and LIN, respectively. Figure S2: Begg's funnel plots of the publication bias. (A–K) Funnel plots of semen volume, sperm concentration, total sperm count, normal morphology rate, progressive motility, total sperm motility, DFI, VCL, VSL, and LIN, respectively.
