# Supplementary material for: Impacts of Outdoor Air Pollution on Human Semen Quality: A Meta-Analysis and Systematic Review
Source: Biomed Res Int. 2020 Apr 28;2020:7528901. doi: 10.1155/2020/7528901 (PMC7204269; doi:10.1155/2020/7528901)

Supplement materials

Figure S1. Sensitivity of each included study in this meta-analysis. A-K indicated sensitivity analyses of semen volume, sperm concentration, total sperm count, normal morphology rate, progressive motility, total sperm motility, DFI, VCL, VSL and LIN respectively.

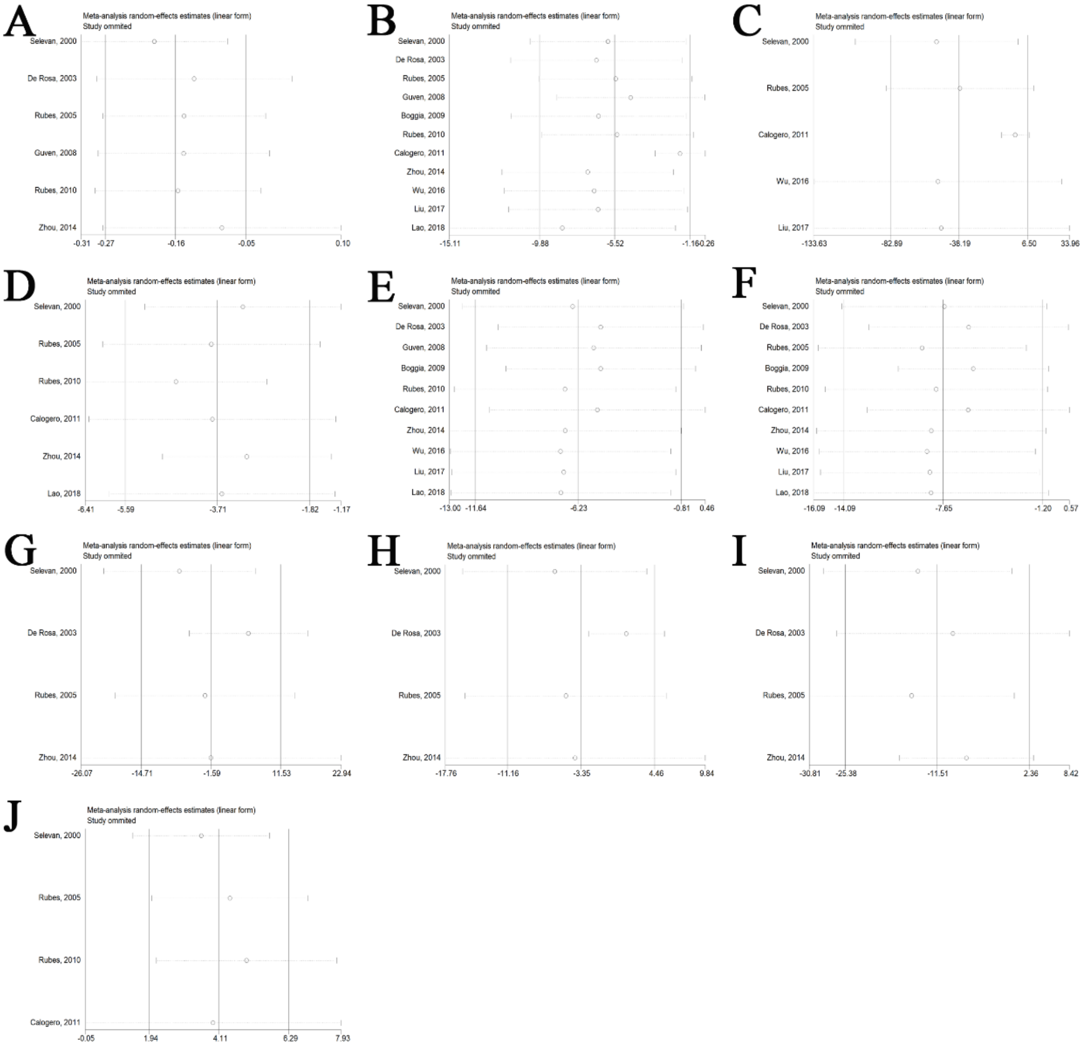

Figure S2. Begg's funnel plots of the publication bias. A-K indicated funnel plots of semen volume, sperm concentration, total sperm count, normal morphology rate, progressive motility, total sperm motility, DFI, VCL, VSL and LIN respectively.

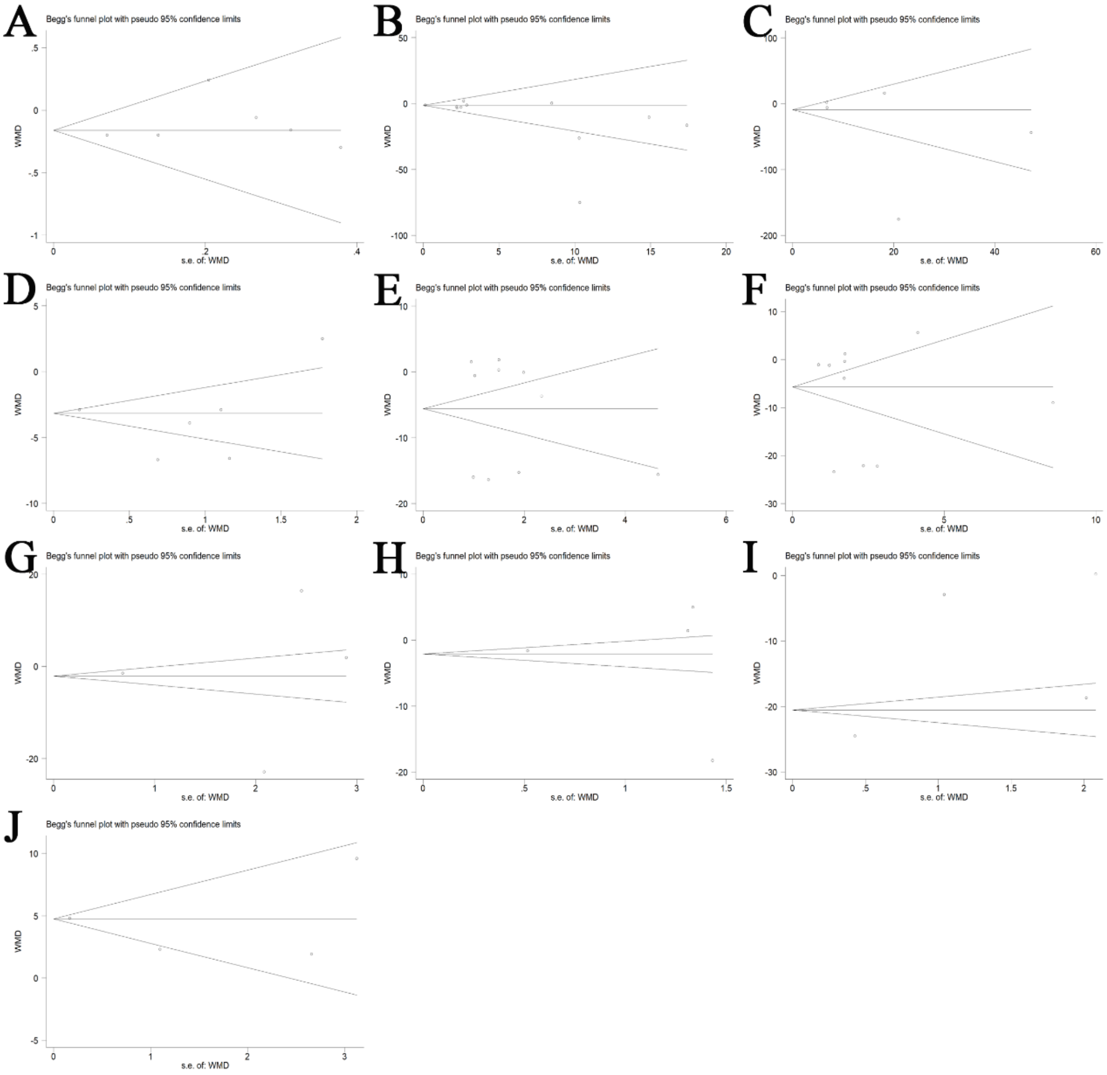

Supplement: Supplementary Materials — Supplement Materials Figure S1: sensitivity of each included study in this meta-analysis. (A–K) Sensitivity analyses of semen volume, sperm concentration, total sperm count, normal morphology rate, progressive motility, total sperm motility, DFI, VCL, VSL, and LIN, respectively. Figure S2: Begg's funnel plots of the publication bias. (A–K) Funnel plots of semen volume, sperm concentration, total sperm count, normal morphology rate, progressive motility, total sperm motility, DFI, VCL, VSL, and LIN, respectively. [file 7528901.f1.pdf]
